# Regulation of male sex hormones strongly heightens copulation behavior indices in rat's model administered *Cocos nucifera* husks extract

**DOI:** 10.4314/ahs.v22i2.47

**Published:** 2022-06

**Authors:** Efosa Bolaji Odigie, Blessing Emosho Atoigwe, Blessing Francis, Monsurat Kehinde Lawal, Theophilus Ogie Erameh, Anthony Nwaobi, Ewamazino Precious Arugba

**Affiliations:** 1 University of Benin, Department of Medical Laboratory Science; 2 University of Benin, Department of Veterinary Physiology & Biochemistry; 3 Igbinedion University Okada, Department of Medical Laboratory Science

**Keywords:** *Cocos nucifera*, coconut husk, Coconut fibre, copulation behaviour, male sex hormones, mating profile, hormonal indices, libido, natural sex enhancer, medicinal values

## Abstract

**Background:**

*Cocos nucifera* (Coconut, Arecaceae family) is consumed as dietary fruit but little is known of it as sex enhancer.

**Objective:**

To investigate male sex hormones and copulation behavior indices in male Wistar rats administered husks extract.

**Materials and Methods:**

Rats were grouped into control A, and treatment B to E (n=4); and administered husks extract at varying doses: 125mg/kg, 250mg/kg, 375mg/kg and 500mg/kg for 48days by oral intubation. Copulation behavior was assessed by introducing female rats to males (1:1) in a rectangular Plexiglas chamber and monitored by veterinary Anatomists. Blood samples for male sex hormones were collected and assayed by ELISA method.

**Results:**

Copulation behavior (500mg/kg treatment): mounting (9.66±0.79 to 29.08±2.16), ejaculation (7.66±0.38 to 16.93±0.76), intromission (22.01±1.67 to 38.11±3.35) frequencies, and ejaculation latency (7.92±0.43 to 12.28±0.41) increased remarkably (Group E). Correspondingly, mounting (133.31±1.18 to 93.39±0.43) and, intromission (88.13±3.12 to 74.55±1.19) latencies; post-ejaculation (3.16±0.14 to 2.18±0.34), inter-intromission (19.48±0.26 to 14.32±1.65) intervals reduced significantly (P≤0.05). Testosterone levels (Group E) increased: 3.82±0.6ng/dL to 5.14±0.3ng/dL while, LH and FSH values reduced: 2.92±0.6IU/L to 2.13±0.3IU/L and 2.28±0.7IU/L to 1.58±0.3IU/L significantly (p ≤ 0.05) while changes were dose-related.

**Conclusion:**

*C. nucifera* husks extract improves sexuality indices by regulating male sex hormones in male Wistar rats.

## Introduction

*Cocos nucifera* is known globally as Coconut from *Arecaceae* or palm family with a subfamily *Cocoideae*. It is commonly consumed as dietary fruit due to its nourishments and refreshing taste. Many people irrespective of race, tribe and colour consume it for different purposes and often as a gonadotrophic agent^1^. The word ‘coconut’ can refer to the whole coconut palm, seed, husk or fruit, which is a drupe botanically as documented but mostly referred to as a nut. Coconut as a name is ambiguous and has completely deviated from the actual connotation of being a drupe, however it may be referred to as a fruit, nut or seed because it is categorized as a fibrous one seeded drupe going by its loosed definitions 2.

The use of plants, plant derivatives or natural products in stimulating sexual arousal, enhancing performances and or increasing libido is as old as human race itself 3. Poor sexual behavior in majority of men has been without headway resulting to breakages in many marital affairs. Overtime, efforts have been made to bring a lasting solution to homes ravaged by poor sexuality, and have ended in futility. Going by vast sexual benefits thought to be attributed to intake of coconut or component parts, sufferers of poor sexuality are advised to increase coconut consumption believing that it will proffer solutions to weak sexual behavior in men 4.

Cocos nucifera husk fibre has been used for oral hygiene, teeth whitening and against oral pathogens, which has proven medically to possess antimicrobial activities among rural dwellers in southern India province 5. Traditionally, it is used in the treatment of cancerous cells, hyperglacemia, stooling and diarrhea, etc. while most claims suggest that it scientific backings are drawn from in-vitro and in-vivo biological evaluations 6. Most people think that husk fibre has no medical importance, which is not in tandem to the usage for treating numerous inflammatory diseases 7. Hexane fraction of coconut husk contained anti-malignant properties but has not been fully harnessed 8. Hypoglycaemic effect has been studied using allozan-induced diabetic rats as model 6. Component parts of *C. nucifera* have been used in the management of varying health challenges and majorly act as cardioprotective, antioxidant antithrombotic, hepatoprotective and antiprotozoal 9. As a natural product, *C. nucifera* has been used as an insect repellant and disinfectant 9.

From the foregoing, it is obvious that *C. nucifera* husks as an aphrodisiac is yet to be studied whether as sex enhancer or for treating low libido in men. This study is novel as far as we are aware, as most literature we came across majorly focused on coconut flesh or milk for sexual purposes. It is against this backdrop that we investigated the influence of *C. nucifera* husks extract on male sex hormones and copulation behavior parameters: mounting, intromission, and ejaculating latencies, post-ejaculation and inter-intromission intervals as well as mounting, ejaculating and intromission frequencies respectively; which collectively determine copulation conduct in animal model 4.

## Materials and Methods

### Collection and extraction of plant materials

Fully developed fresh green coconut palm fruits (*C. nucifera*) were harvested from a coconut palm tree at Ekoshodi community behind the University of Benin, Benin City; Nigeria. They were identified and authenticated by a taxonomist and assigned a voucher number (V.1041/78) before depositing a sample in the herbarium. The Coconut were carefully de-husked with a cutlass, washed with running tap water to expel dirt, slit into pieces, and shade dried at room temperature for 14 days. A household electric blender (Kenwood 1.6L, BL480 Prestons, Australia) was used in grinding the dried husk fiber for 5 minutes. The rough blend was repeated until a uniform powder was observed and filtered with a kitchen plastic filter. Extract from powdery fibrous husk (1,290.25g) was separated in a Soxhlet apparatus with 1.5L of ethanol as solvent, and concentrated using a rotary-evaporator^10^. Extract was dissolved in an aqueous solution of varying concentrations: 125mg/kg, 250mg/kg, 375mg/kg and 500mg/kg whenever it was needed.

### Selection and preparation of experimental animals

Twenty (20) wistar rats (Rattus norvergicus, 9–13weeks old, average weight: 162.3g, range 146.2g - 178.6g) obtained from the animal house, Department of Animal and Environmental Biology, University of Benin were used for this study. We used a hygienic metallic gauze cages lined with saw-dust as duvets to curtail animals in a highly aerated apartment where the rats were harbored from birth. They however adapted for 7days with ambient temperature of (24°C) ± 5, humidity of 50–55% and photoperiod of 12:12 hours light/dark cycle. Rats were provided with standard feeds pellets (Growers mash, Vital Feed, Grand Cereal, Nigeria) and drinking water ad libitum. This study conformed to lay down rules and regulations for protecting the rights of animals used in experimental designs 11. We followed the guideline document on humane and endpoints for conducting toxicity check on experimental rats so as to reduce their sufferings 12, and evaluation of natural medicine promulgated by W.H.O 13.

### Method of extract administration

Rats were selectively arranged into five (5) groups according to their weight termed: A to E containing 5 male rats per group. Rats in group A (control) were given distilled water while group (B to E) received doses of extract calculated in mg/kg: 125mg/kg, 250mg/kg, 375mg/kg and 500mg/kg and administered once daily by oral intubation for 48days with orogastric tube.

### Acute toxicity study

Toxicity studies for coconut husk extract were determined using modified Lorke's method 14. In this phase, sixteen rats were selected into three groups and a control (n=4; two males and two females per group). Extrapolated doses (375 mg/kg, 750 mg/kg and 1,225 mg/kg) were administered daily for six weeks. All rats were closely monitored particularly in the first few hours for abnormal displays followed by intermittent watch for 24hr.

### Cage side observation

Both treated and control rats were monitored on daily basis for abnormalities or behavioral signs of acute toxicity throughout the study period. In addition, animals were closely observed after treatment for activities like whizzing, climbing, anticipation, licking, sniffing, bouncing e.t.c

### Empirical measurements (Organ and Body)

Internal genitals: testes, epididymis, seminal vesicle and prostate gland were weighed at the end of the experiment and compared with the controls; while all rats were weighed before and after treatment regimen. A digital electronic weighing balance sensitive to 0.001g (Gilbertini, Italy) was used for this purpose. Differences in weight were calculated in grams by subtracting final weight from initial while, amount of feeds consumed in a day was documented in g/day.

### Pre-copulation testing

Male rats were subjected to pre-copulation testing to identify animals that are not sexually active before administration of extracts. They were trained for sexual activities by introducing female rats into the observation chamber twice daily for 7 days.

### Copulation Behavioral Tests

Before introducing female rats for copulation, behavioral activities showing that animals are on heat was closely monitored in male rats, which included anogenital snuffling and genital grooming. Investigation was conducted on 16^th^, 32^nd^ and 48^th^ days after consuming *C. nucifera* husks extract 15. Veterinary Anatomy expert observers that were blinded to the experimental design in a soundproofed environment monitored the animals during the dark phase of the light/dark cycle. A single male rat was placed in a rectangular Plexiglas monitoring chamber measuring (45cm×40cm×30cm) and allows acclimating with the cage environment for 7minutes. Later on, primed female rats that were nulliparous and non-pregnant were introduced to the males (1:1). Copulation behavior such as female attractions, withdrawals from opposite sex, closeness and or caressing was examined after introducing female rats 15. Active exploration behaviors known as pre-copulation factors like locomotion, sniffing and rearing were monitored in the first few minutes before copulation started. However, copulation was repeated 10minutes after end of each test particularly when there was no ejaculation within an exhaustive period.

### Copulation behavior parameters

The following copulation parameters were documented such as mounting latency (ML), which was observed as the time from introduction of the female until the manifestation of the first mount. Intromission latency (IL) was observed as the time that the female was first introduced to the time of first vaginal penetration. Ejaculating latency (EL) was observed as the time from first vaginal penetration (intromission) till animal attained ejaculation. Mounting frequency (MF) observed as the number of consecutive mounts prior to ejaculation. Post-ejaculating interval (PEI) observed as the time from ejaculation till the following vaginal penetration (intromission). Ejaculating frequency (EF) calculated as the total number of ejaculations within 30min intervals 16. Intromission frequency (IF) noted as the number of vaginal penetrations (intromissions) before ejaculating. Inter-intromission interval (III) showed as average intervals between successive vaginal penetrations (intromissions). Note: ML, IL and III were calculated in seconds, PEI and EL in minutes while MF, EF and IF in numbers of occurrence.

### Blood samples collection

About 1ml of blood was collected from the marginal ear vein of each rat (test and control) with a 2ml needle and syringe for hormonal assay before commencing treatment (day 0). It was repeated in all animals, during the treatment (24th day of administration). After the experiment (day 48), all rats fasted overnight to the 49th day and were euthanized via decapitation. After sacrificing the animals, close to 1.5ml of blood was collected via cardiac puncture to determine final analysis for hormonal assay of experimental rats. Blood sample was collected from animals into plain test tubes between 7am and 9am whenever collection was done.

### Analysis for male sex hormones

All blood samples (before, during and after experimentation) were centrifuged at 2500 rpm for 5 minutes using centrifuge machine (MSE 846307 England). Sera were micro auto-pipetted and aseptically emptied into sterile vials stored at -20oC till they were used to determine the levels of testosterone (Te), follicle stimulating hormones (FSH), and luteinizing hormones (LH) respectively. Enzyme-linked immunosorbent assay (ELISA) kit for Testosterone (Crystal Chem Inc. USA) and Elabscience Biothecnology Ltd. China for FSH and LH were used according to manufacturer's instruction.

### Grossing, tissue processing and histopathology

After sacrificing all male rats, internal genitals (testes, epididymis, seminal vesicle and prostate gland) were dissected, harvested, weighed with a précised electronic digital balance and grossed. Testes were fixed in bouin's fluid while epididymides, seminal vesicles and prostate glands were fixed in 10% neutral buffered formalin (NBF). Tissues were processed with automatic tissue processor (Hestion -ATP7000 tissue processor-Germany), and embedded using digital embedding machine (Hesition-E500 Germany). Sections were cut at 5µm thickness using digital rotary microtome (Hestion ERM 4000 Germany). They were heat fixed onto frosted end glass slides and stained with hematoxylin and eosin in preparation for light microscopy. Slides were examined for pathological abnormities and blindly reviewed by pathologists using Swift Binocular compound light microscope® (Olympus England) with Photomicrograph (Leica ICC50, China) under X400 full magnification.

### Statistical Analysis

IBM SPSS version 20.0 was used to carry out the statistics Data were analyzed using ANOVA) while results were presented as means ± S.E.M (standard error of mean). Turkey's post-hoc was used where the assumption of homogeneous of variance was assumed for pairwise comparison between groups. Values with the same superscript were not statistically significantly different while the values with difference in superscript were considered significantly different as p-value was set at 0.05.

## Results

No signs of acute toxicity, morbidity and or mortality were observed throughout the study; showing that coconut husk is non-lethal, and tolerable with considerable safety margin.

**Figure 1:**
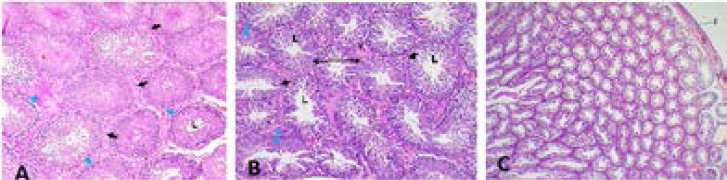
**A** = Control section of left testes revealed seminiferous tubules with lumen (L) mostly filled with spermatids (Blue Arrows = spermatogonia, one of the layer of germinal epithelia), surrounded by interstitial connective tissues (Black Arrows). **B** = Section of left testes treated with high dose (375mg/kg) of husks extract and is in keeping with normal histology. It showed seminiferous tubules (Double Edged Black Long Arrow) with lumen (L) mostly filled with spermatids, and layers of germinal epithelia (Double Edged Blue Short Arrows). **C** = Section of left testes treated with highest dose (500mg/kg) of husks extract with normal histology. It showed seminiferous tubules with conspicuous lumen, with tunica albuginea and blood vessels marked (T /V). All sections stained with haematoxylin and eosin at X400 magnification.

**Figure 2:**
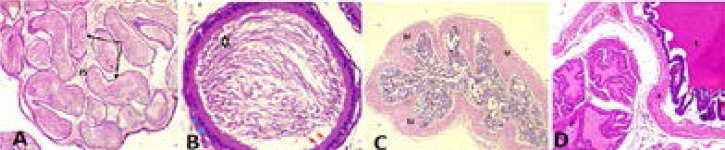
**A** = Control section of left epididymis revealed fibromuscular stroma (FS) with conspicuous numbers of epididymis containing mature sperm cells (Black Long Arrow). **B** = Section of left epididymis treated with 100mg/ml of husks extract is with normal histology. Section revealed mature sperm cells - spermatozoa (Black Star) surrounded by basal cells (Blue Arrow) and strereocillia (Red Arrow). **C** = Control section of seminar vesicle revealed lumen (L) filled with acidophilic secretions, and mucosa fold showing very little secretion (Red Arrow) including muscularis marked (M). **D** = Section of seminar vesicle treated with 500mg/kg of hurks extract is with normal histology. It showed lumen (L) filled with acidophilic secretions and trabeculi marked (#). Sections stained with haematoxylin and eosin at X400 magnification.

**Figure 3:**
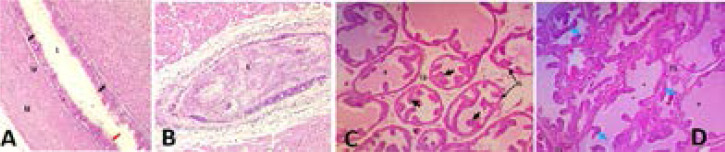
**A** = Control (longitudinal) section of Vas deferens revealed lumen (L), lined by pseudostratified epithelium (Double Edged Black Short Arrow) and strereocillia (Red Arrow) surrounded by Muscularis (M). B = Longitudinal section of Vas deferens treated with 500mg/kg of husks extract (normal histology). It showed lumen (L) filled with mature sperm cells, and lined by pseudostratified epithelium. C = Control section of Prostate revealed prostate gland acini marked (A), papillary infolding of acinal lining (Black Arrow), eosinophilic secretion (Asterisks) and fibromuscular stroma marked (FS). D = Section of Prostate treated with 500mg/kg of husks extract (normal histology). It showed fibromuscular stroma (FS) with eosinophilic secretion marked (Asterisks) and lined by cuboidal to columnar epithelial lining (Blue Arrow). Sections stained with haematoxylin and eosin at X400 magnification.

Average body weight of rats was 162.4g with an average food consumption of 10.2±1.7g/day. Body weight of treated rats reduced and demonstrated dose dependent decreases compared to controls with elevated values (6.53 ± 0.3g↑↑). Moderate reductions were attributed to low dose treated rats: 125mg/kg, Group B (1.46 ± 0.9g↓) and 250mg/kg, Group C (2.65 ± 0.4g↓) while, weight of animals decreased remarkably as dosage increases: 375mg/kg, Group D (5.72 ± 1.5g↓↓); 500mg/kg, Group E (6.77 ± 2.8g↓↓). Rat's feeds consumed also reduced on daily basis just as treated animals lost weight corresponding to increments in dosage (Table 1). Physical activities were observed in all treated rats without signs of dullness, anxiety, sleeplessness, or restlessness and particularly in 500mg/kg extract treatment (Table 1). There were no marked differences in organ weights between treated and untreated animals (Table 2). Histology of internal genitals: testes, epididymis, seminar vesicle, Vas deferens and prostate both treated or control did not reveal any pathological lesions after 48days of daily oral consumption of husks extract (Figure 1 to 3).

**Table 1:** Weight changes, Activities and Feeds in Male Rats Administered *C. nucifera* Husks Extract

Groups	Doses (mg/kg)	Initial mean weight (g)	Final mean weight (g)	Difference in weight	Physical Activities	Food intake g/day
A	0	150.14 ± 1.4	156.67 ± 2.2**	6.53 ± 0.3↑↑	+	14.6 ± 1.7
B	125	150.67 ± 1.8	149.21 ± 4.7	1.46 ± 0.9 ↓	+	11.4 ± 2.2
C	250	160.38 ± 2.9	157.73 ± 2.6	2.65 ± 0.4 ↓	+	10.3 ± 1.2
D	375	170.14 ± 2.6	165.42 ± 2.7*	4.72 ± 1.5 ↓↓	+	7.5 ± 2.6
E	500	176.11 ± 3.3	170.34 ± 3.8**	5.77 ± 2.8 ↓↓	++	7.2 ± 1.9

All values are expressed as mean ± standard error of the mean.

Distilled water was administered to untreated rats representing 0mg/ml. Symbols were used to assess and conote physical activities: negligible (±); present (+); negligible increase (↛); moderate increase (→); highly present (++); absent (-); negligible decrease (↓̸); moderate decrease (↓); remarkable decrease (↓↓); remarkable increase (↑↑).

**Table 2:** Effect of Varying Concentrations of Extract on Organ Weight In Experimental Rats

Organs	Group A (control)	Group B (125mg/kg)	Group C (250mg/kg)	Group D (375mg/kg)	Group E (500mg/kg)	P-value
Left Testis	0.740 ± 0.03	0.748 ± 0.03	0.749 ± 0.16	0.752 ± 0.91	0.752 ± 0.48	0.106
Right Testis	0.742 ± 0.28	0.746 ± 0.67	0.731 ± 0.09	0.746 ± 0.08	0.748 ± 0.31	0.163
Left Epididymis	0.506 ± 0.29	0.497 ± 0.14	0.493 ± 0.64	0.480 ± 0.04	0.501 ± 0.21	0.016
Right Epididymis	0.226 ± 0.23	0.212 ± 0.13	0.159 ± 0.34	0.190 ± 0.08	0.191 ± 0.53	0.265
Left Seminal gland	0.094 ± 0.16	0.125 ± 0.35	0.126 ± 0.87	0.130 ± 0.01	0.130 ± 0.43	0.001
Right Seminal gland	0.088 ± 0.17	0.112 ± 0.09	0.088 ± 0.91	0.124 ± 0.08	0.125 ± 0.12	0.004
Prostate gland	0.278 ± 0.13	0.283 ± 0.05	0.229 ± 0.04	0.316 ± 0.05	0.316 ± 0.19	0.012

All values expressed as Mean ± standard error of the mean. P-value is set at 0.05

Hormonal analysis of both treated and untreated rats; Te, LH and FSH levels before treatment were in the normal range (Table 3). A significant increase in the level of Te (p ≤ 0.001) and marked decreases of LH and FSH (p ≤ 0.05) during experimentation were notable (Table 4). At termination of the experiment (Table 5), testosterone level in Group E was further increased: before (3.82 ± 0.6ng/dL), during (4.92 ± 0.5ng/dL), and after treatment with 500mg/kg of husks extract (5.14±0.3ng/dL). The results in Group E also showed a corresponding decrease in values of LH: 2.92 ± 0.6IU/L; 2.22 ± 0.5IU/L; 2.13 ± 0.3IU/L including FSH: 2.28 ± 0.7IU/L; 1.77 ± 0.7IU/L; 1.58 ± 0.3IU/L respectively and were significant across board (p ≤ 0.05).

**Table 3:** Hormonal Analysis of Male Albino Rats on Day Zero (0) Prior to Experimentation

Dosage	T (ng/dL)
Control	3.32 ± 0.3
125mg/kg	3.18 ± 0.1^b^
250mg/kg	2.79 ± 0.3^b^
375mg/kg	3.50 ± 0.5^a^
500mg/kg	3.82 ± 0.6^a^
P-value	0.011

All values are expressed as mean ± standard error of the mean. Values in the same column with the same superscript significantly varied at P ≤ 0.05 (ANOVA) on pairwise comparison. Testosterone (Te), Follicle stimulating hormones (FSH), Luteinizing hormones (LH).

**Table 4:** Hormonal Analysis of Male Albino Rats on Day 24 (Midway) of Experimentation

Dosage	Te (ng/dL)	LH (IU/L)	FSH (IU/L)
Control	3.22 ± 0.7	2.79 ± 0.6	2.09 ± 0.1
125mg/kg	4.13 ± 0.3c	2.72 ± 0.7^a^	1.43 ± 0.1^b^
250mg/kg	4.32 ± 0.3c	2.66 ± 0.1^a^	1.57 ± 0.5^b^c
375mg/kg	4.77 ± 0.4^b^	2.59 ± 0.3^b^	1.66 ± 0.3c
500mg/kg	4.92 ± 0.5c	2.22 ± 0.5^b^	1.77 ± 0.7c
P-value	0.023	0.013	0.001

All values are expressed as mean ± standard error of the mean.

Follicle stimulating hormones (FSH), luteinizing hormones (LH), Values in the same column with the same superscript significantly varied at P ≤ 0.05 (ANOVA) on pairwise comparison

**Table 5:** Hormonal Analysis of Male Albino Rats on Day 49 (After) Experimentation

Dosage	Te (ng/dL)	LH (IU/L)	FSH (IU/L)
Control	3.03 ± 0.1	2.92 ± 0.2	2.03 ± 0.6
125mg/kg	4.32 ± 0.5^b^	2.81 ± 0.4^a^	1.66 ± 0.1c
250mg/kg	4.61 ± 0.7^b^	2.43 ± 0.6^a^	1.56 ± 0.3^b^
375mg/kg	4.73 ± 0.4^b^	2.22 ± 0.2^b^	1.74 ± 0.2^b^
500mg/kg	5.14 ± 0.3c	2.13 ± 0.3^b^	1.58 ± 0.3^b^
P-value	0.001	0.012	0.011

All values are expressed as mean ± standard error of the mean. Values in the same column with different superscript significantly varied at P ≤ 0.05 (ANOVA) on pairwise comparison

Pre-mount activities in this study were more of climbing, licking, anogenital sniffing and grooming genitally. It was often observed that male rats advanced towards their female counterparts (1:1) in an erratic manner. The animals were full of life and expectations, while the aforementioned modes of appearance were peculiar with experimental rats, and were increasingly noticeable on 32nd and 48th days of treatment compared to 16th day and untreated animals. In the 16days post exposure, parameters: MF, EF, IF, and EL increased after consuming husks extract (Table 6). Results showed striking and progressive increments particularly in IF from 125mg/kg treatment, Group B (12.22 ± 1.32) to 500mg/kg treatment, Group E (22.01 ± 1.67) in contrast to untreated rats (9.97 ± 0.68), which indicated that elevated values were dependent on dosage (p = 0.028). Mounting frequency (MF) was also seen to have risen from (4.89 ± 0.27 to 9.66 ± 0.79) but was not significant statistically (p = 0.063). Correspondingly, ML, III, and IL decreased significantly (p ≤ 0.05) while PEI (4.54 ± 0.23 to 3.16 ± 0.14) reduced singularly but was statistically insignificant (p = 0.133).

**Table 6:** Copulation behavior in male rats on 16^th^ day of experimentation (exposure for 16 days)

Copulation Parameters	Control	125mg/kg	250mg/kg	375mg/kg	500mg/kg	P-value
Mounting latency (ML) sec.	276.11 ± 2.33	181.54 ± 2.02	157.99 ± 1.14*	148.58 ± 1.43*	133.31 ± 1.18**	0.037
Mounting frequency (MF)	3.92 ± 0.11	4.89 ± 0.27*	5.58 ± 0.77*	8.36 ± 0.94*	9.66 ± 0.79*	**0.063**
Ejaculating frequency (EF)	2.62 ± 0.17	3.09 ± 0.15	5.07 ± 0.42*	6.63 ± 0.55*	7.66 ± 0.38**	0.041
Intromission latency (IL) sec.	118.24 ± 3.27	112.52 ± 1.88*	101.33 ± 1.66**	96.03 ± 1.92**	88.13 ± 3.12**	0.027
Intromission frequency (IF)	9.97 ± 0.68	12.22 ± 1.32*	16.19 ± 1.25*	18.73 ± 1.56*	22.01 ± 1.67**	0.028
Ejaculating latency (EL) min.	4.58 ± 0.46	5.28 ± 0.44*	6.16 ± 0.22*	6.78 ± 0.21*	7.92 ± 0.43*	**0.145**
Post-ejaculating interval (PEI) min.	5.16 ± 0.32	4.54 ± 0.23*	4.01 ± 0.13*	3.75 ± 0.02*	3.16 ± 0.14*	**0.133**
Inter-intromission interval (III) sec.	39.63 ± 0.52	28.79 ± 1.84*	27.26 ± 1.33*	22.55 ± 4.41**	19.48 ± 0.26**	0.021

All values are expressed as mean ± standard error of the mean for 4 rats per group Values with different asterisks as superscript in the same row significantly varied at P ≤ 0.05 (ANOVA) on pairwise comparison Calculation for ML, IL and III in seconds, PEI and EL in minutes, while MF, EF and IF in numbers of occurrence

Table 7 showed that there were improvements in elevated values: MF (5.27 ± 0.14 to 13.88 ± 1.32), EF (5.14 ± 0.13 to 13.71 ± 0.63), IF (16.01 ± 1.26 to 32.82 ± 2.63) and EL (6.88 ± 0.44 to 11.93 ± 0.05) compared to controls (3.96 ± 0.92; 2.77 ± 0.36; 9.56 ± 0.53; and 4.35 ± 0.16). Despite the obvious rise in value, EL was insignificant statistically (p=0.331) while others: MF, EF, and IF were highly significant (p= 0.011; 0.026 and 0.023) one-to-one. Contrarily, ML (159.16 ± 1.02 to 121.01 ± 1.82) and IL (102.44 ± 2.98 to 83.96 ± 2.88) including PEI (4.21 ± 0.45 to 2.83 ± 0.52) and III (25.03 ± 1.72 to 16.23 ± 1.78) all decreased in response to increasing administered dosage compared to control (Table 7). Post-ejaculating interval (PEI) did not decrease significantly (p = 0.065) while other parameters (ML, IL, and III) significantly reduced in favor of this investigation (p= 0.033; 0.043; and 0.001). A striking result was obtained in the third phase of this study (hush fibre treatment from day 33 to 48), and is suggestive of a dose related upward and or downward review of copulation parameters. As usual, from 125mg/kg to 500mg/kg, parameters either increased: MF (6.93 ± 0.77 to 29.08 ± 2.16), EF (7.77 ± 0.25 to 16.93 ± 0.76), IF (19.93 ± 1.87 to 38.11 ± 3.35), and EL (8.65 ± 0.51 to 12.28 ± 0.41) and decreased: ML (141.41 ± 2.96 to 93.39 ± 0.43), III (22.36 ± 1.34 to 14.32± 1.65), IL (99.66 ± 2.78 to 74.55 ± 1.19), and PEI (4.11 ± 10.55 to 2.18 ± 0.34) where necessary compared to controls (Table 8). Values were however significant statistically (P ≤ 0.05) excluding EL (p = 0.179). Also, there was a progressive and consistent rise in values across all groups of treated animals (Table 8). Copulation behavioral experimental days (16th, 32nd and 48th) were compared with the paired t-test and recorded a significant value (p ≤ 0.045).

**Table 7:** Copulation Behavior In Male Rats On 32^nd^ Day of Experimentation (Exposure for 32 days)

Copulation Parameters	Control	125mg/kg	250mg/kg	375mg/kg	500mg/kg	P-value
Mounting latency (ML) sec.	273.02 ± 1.64	159.16 ± 1.02	147.11 ± 1.21*	142.91 ± 1.33*	121.01 ± 1.82**	0.033
Mounting frequency (MF)	3.96 ± 0.92	5.27 ± 0.14	7.35 ± 0.19*	9.06 ± 1.91**	13.88 ± 1.32**	0.011
Ejaculating frequency (EF)	2.77 ± 0.36	5.14 ± 0.13*	6.03 ± 0.21*	9.86 ± 0.22**	13.71 ± 0.63**	0.026
Intromission latency (IL) sec.	121.83 ± 2.23	102.44 ± 2.98	93.66 ± 3.76*	86.52 ± 1.19*	83.96 ± 2.88*	0.043
Intromission frequency (IF)	9.56 ± 0.53	16.01 ± 1.26*	19.11 ± 1.67*	24.77 ± 1.28**	32.82 ± 2.63**	0.023
Ejaculating latency (EL) min.	4.35 ± 0.16	6.88 ± 0.44*	6.93 ± 0.17*	9.47 ± 0.25**	11.93 ± 0.05**	**0.331**
Post-ejaculating interval (PEI) min.	5.15 ± 0.29	4.21 ± 0.45*	3.83 ± 0.66*	3.32 ± 0.19*	2.83 ± 0.52**	**0.065**
Inter-intromission interval (III) sec.	40.82 ± 2.31	25.03 ± 1.72**	23.11 ± 2.11**	19.87 ± 1.66**	16.23 ± 1.78**	0.001

All values are expressed as mean ± standard error of the mean for 4 rats per group

Values with different astericks as super script in the same row significantly varied at P ≤ 0.05 (ANOVA) on pairwise comparison

Calculation for ML, IL and III in seconds, PEI and EL in minutes, while MF, EF and IF in numbers of occurrence

**Table 8:** Copulation Behavior In Male Rats On 48^th^ Day of Experimentation (Exposure for 48 days)

Copulation Parameters	Control	125mg/kg	250mg/kg	375mg/kg	500mg/kg
Mounting latency (ML) sec.	274.23 ± 3.48	141.41 ± 2.96*	122.38 ± 2.12*	101.05 ± 1.67*	93.39 ± 0.43***
Mounting frequency (MF)	3.88 ± 0.64	6.93 ± 0.77*	10.28 ± 1.54**	17.11 ± 1.66**	29.08 ± 2.16***
Ejaculating frequency (EF)	3.06 ± 0.91	7.77 ± 0.25**	10.86 ± 0.11**	13.04 ± 0.52***	16.93 ± 0.76***
Intromission latency (IL) sec.	124.83 ± 9.23	99.66 ± 2.78*	86.24 ± 1.45**	81.37 ± 0.75**	74.55 ± 1.19***
Intromission frequency (IF)	9.88 ± 0.53	19.93 ± 1.87*	21.39 ± 1.55**	28.01 ± 1.63***	38.11 ± 3.35***
Ejaculating latency (EL) min.	5.25 ± 0.13	8.65 ± 0.51*	8.71 ± 0.54*	11.16 ± 0.03**	12.28 ± 0.41**
Post-ejaculating interval (PEI) min.	5.44 ± 0.73	4.11 ± 10.55*	3.12 ± 0.10*	2.64 ± 0.02**	2.18 ± 0.34**
Inter-intromission interval (III) sec.	39.82 ± 0.66	22.36 ± 1.34**	19.24± 1.12**	16.01± 2.49**	14.32± 1.65***

All values are expressed as mean ± standard error of the mean for 4 rats per group

Values with different asterisks as super script in the same row significantly varied at P ≤ 0.05 (ANOVA) on pairwise comparison

Calculation for ML, IL and III in seconds, PEI and EL in minutes, while MF, EF and IF in numbers of occurrence

## Discussion

Coconut has been said to possess different kinds of beneficial properties such as vitamins, sugar, protein, electrolytes, minerals, dietary fibres, antioxidants, cytokins e.t.c, and has been particularly demonstrated in human's health^17^. However, little information is available on the pathological point of view and particular as it is often used for fertility purposes while undermining a potential harm to internal male genitalia. Surprisingly, we did not record any deleterious effects exhibited by consumption of coconut husks even in large quantity (500mg/kg, Group E), which is comparable with the reports of other researchers 6, 18, 19. This study is in tandem with Costa et al.^20^ that reported positive influences of coconut on male rat's sex organs with no harm to the histology.

The present study advises that extract drink has high influence on appetite by reducing cravenness for food. This alone contributed massively to reduced body weight loss in experimental rats, which showed obvious reductions in feeds consumed by animals. Though feeding habit reduced in rats (Table 1) it can be said that this observation is dose related as the reductions occurred progressively as dosage increases. This pattern of body weight control in animal model has been reported 19. It is however not unconnected to a decrease in catabolic activity in treated animals while untreated experienced increases in catabolism, which supports scientific reports that food intake increases catabolic activity in the body, and in turn has potential effects on body' systems 21.

Effect of husk extracts on sex hormones are laudable in this study going by results obtained prior to experimentation compared to values seen during and after treatments. Testosterone levels increased magnificently in the last phase of testing; particularly in high doses as against the same prior to treatment suggesting that *C. nucifera* hush can restore poor testosterone to normal. This report however agrees with the suggestion that obesity highly influences reduction in testosterone including other factors like diabetics mellitus, which is partly responsible to reduced sexual activities 22, 23. Also, high testosterone level combined with a striking balance in FSH and LH is needed to effectively maintain better sexual performance and erotic displays in men, which has been reported to be responsible for normal spermatogenesis possibly regulated by the hypothalamic system 24. In another development, Oyovwi et al.^5^ examined the effects of *C. nucifera* extract on hypothalamo-pituitary-gonadal axis and fertility in rats and obtained a significant regulation in LH and FSH, including testosterone levels respectively. The result was attributed to increased testicular diameter and weight including increased epididymal and interstitial sizes. The mechanism of action in increased testicular weight is presumed to be due to stimulation of receptors in testis for growth hormone 26.

In this study, pre-copulation testing of both test and control animals were found to be less sexually active. However, after treatment with husks extract copulation behavior parameters in male rats suggest that the extracts contribute positively to vigour and libido, and is highly suggestive of erectile effectiveness, penile coordination and ejaculating reflexes. It has been reported that decreased (ML, III, IL, and PEI) and or increased (MF, EF, IF, and EL) in copulation parameters collectively determine how best sexual performances are enhanced^27^. Sex enhancement substance is said to be potent and acceptable for men's usage if only it can regulate copulation parameters as indicated earlier1. These factors have been shown to represent increased libido, ejaculating reflexes, and vigor in men^4^, ^16^. The present study is in tandem with a lot of investigations suggesting that elevated (MF, EF, IF, and EL) and reduced (ML, III, IL, and PEI) signify sexual improvements 27. This study suggests that husk extract enhances sexual drive, erectile efficiency and penile orientation as it was found to have resulted in an increase in ML, III, IL, and PEI from the lowest dose during the first 14 days of administration (Table 5). It was further observed to have improved spontaneously as days of administration increased from the lowest to the highest administered doses on 15^th^ to 32nd days of treatment (Table 6). The improvements were however continuous and progressive through the last phase of husk treatment between day 33 to 48 respectively (Table 7). The reverses were the case with respect to decreasing values: ML, III, IL, and PEI, and have been reported in scientific literatures 1.

Ability to boost sexual energy is not unconnected to phytochemicals and their metabolites widely available in the different parts of Coconut, and may contribute to boosting sexual performances in men 1, 28. Phenolic compounds and flavonoids have been reported in husks extract, which further aid stimulation of antioxidant activities, thereby improving penile and erectile malfunctioning, restoring loss of libido and enhancing sexual ability 19, 29. In a related study, Relebona et al.^30^ observed increased and decreased values in parameters reported in this study. Although, the former 30 worked on *G. kola* while we studied *C. nucifera* husks fibre. Fouche et al.^16^ reported that elevated MF is particularly alleged to be frontiers for assessing libido, potency, and penile reflexes, which therefore affirmed that a rise in value is strongly suggestive of being able to contribute positively to sexuality in human. Prakash et al. 1 argued that higher MF is attributed to libido, potency and penile coordination. We however, proposed that MF supports effectiveness in erection, ejaculation reflexes and vigor as these words are seen to be ambiguous and often used interchangeably with the former 1, 16 but not entirely the same. In line with the ongoing, animals that gained weight excessively (controls) had a reduced copulation performance indices compared to treated rats that showed controlled body weights in this study. Hence, it can be inferred that body weight control influences the rate of sexual performance, which is synonymous to other findings 22, 23. It has also been argued that obesity contributes largely to testosterone levels while balanced hormonal profile has been attributed to sexual performance^31^.

## Conclusion

*C. nucifera* husks extract in our study is not injurious to histology of internal genitals of male rats. It retains sexual enhancing effect and validates the use of the extract for improving male sexuality. We suggest that improved level of male sex hormones amid body weights control strongly heighten copulation indices in male wistar rats administered husk extract once daily. Further research is advocated to compliment the above claims and to establish safe dose regime.
